# Bistable Networks Enable Complex Shape Changes

**DOI:** 10.1002/advs.76850

**Published:** 2026-07-31

**Authors:** Sawyer Thomas, Jeffrey Lipton

**Affiliations:** ^1^ Department of Mechanical Engineering University of Washington Seattle Washington USA; ^2^ Department of Mechanical and Industrial Engineering Northeastern University Boston Massachusetts USA

**Keywords:** asymmetry, auxetics, bistability, fabrication, frustration, functionally graded material, geometric, metamaterial, state space

## Abstract

The ability to change a surface's profile allows biological systems to effectively manipulate and blend into their surroundings. To mimic this behavior, Mechanical metamaterials can be pre‐programmed during fabrication for complex single deformations. Multi‐stability has enabled metamaterials with programmable mechanical properties and complex shape changes. However, these multi‐stable structures either have a limited number of stable states or no method of achieving the complexity of the profiles available in prefabricated structures from their large state space. Here we show that by coupling bistable elements in a periodic array, we can navigate a vast and otherwise degenerate state space, allowing us to encode targeted and varied shape transformations. We decouple shape programming force from holding force, so low force actuation is amplified into stable and large displacement shape changes. This subset of scale‐independent, additively manufactured metamaterials harnesses shearing to enable asymmetry. They can be automatically rewritten after fabrication to generate complicated 2D profiles and laminated to form 3D surfaces. For successful navigation between profiles with no mechanical frustration, we have developed an inverse shape matching strategy and physically demonstrate the results using an automatic material encoding machine. Our work opens new opportunities in microdevices, tactile displays, manufacturing, and robotic systems.

## Graphical abstract

Transition‐controlled metamaterials are networks of bistable mechanical memory that store local binary states and express them as global shape change. By decoupling low‐force programming from high‐force holding, a single lattice is reconfigured into distinct 2D profiles and 3D surfaces without continuous actuation, enabling reusable morphing materials for robotics and adaptive interfaces.

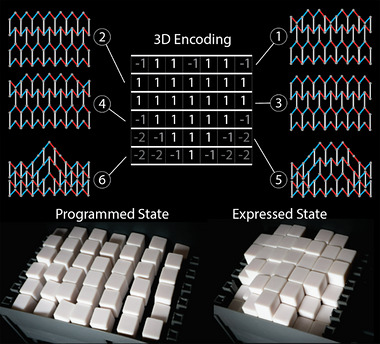

## 1 Introduction

In the natural world, rapid shifts in texture allow animals such as frogs, cuttlefish, and octopi to blend into their surroundings [1, 2], in our own fingertips, the wrinkling of skin improves our ability to grip objects underwater [3]. Birds morph their wing shape to transition between stable and unstable states [4]. These biological examples motivate the broader value of multistable shape‐changing mechanical systems. Our ability to emulate the flexibility of these systems through shape‐transforming techniques has evolved greatly in recent years, but programmatic realization of complex, passively stable morphing remains an issue. We build a new subset of mechanical metamaterials and provide a novel strategy for explicitly navigating between a wide range of desired shapes. This provides reprogrammable and mechanically stable shape states that persist without continuous energy input.

Current morphing techniques can be classified into two primary categories: actuated structures and deforming metamaterials. Actuated surfaces rely on numerous independent actuators to manipulate and continuously maintain their form, which can make some systems bulky and energy‐intensive to hold a static shape [5, 6, 7, 8, 9]. These can be effective for implementations such as stationary haptic displays [5, 7, 9], but are limited in portability and resolution [6]. These morphing systems can employ compact and comparatively fast actuation, including artificial‐muscle, pneumatic, hydraulic/fluidic, electroactive‐material, and shape‐memory actuation systems [10, 11, 12, 13, 14]. However, many such systems require maintained pressure, voltage, temperature, or other active input to hold a commanded form, motivating approaches that preserve a programmed shape as stable mechanical memory. Alternatively, mechanical metamaterials demonstrate additional functionality by tailoring deformation based on architected form. Despite significant advancements, many of these must be programmed at construction, limiting accessible states to a single transformation [15, 16, 17, 18, 19, 20, 21, 22, 23, 24, 25, 26, 27, 28]. While specific materials have dynamically reprogrammable properties [29, 30, 31, 32, 33], explicitly navigating between multiple morphologies without system degeneracy requires a local modulation strategy. Others have shown we can drive material deformation directly, similar to actuated surfaces, but this method results in similar associated challenges [10, 11, 34, 35, 36, 37, 38]. An alternative to direct actuation is to geometrically encode metamaterials at states where low energy barriers separate many deformation paths [39, 40, 41]. For example, in origami‐based metamaterials, this encoding occurs at the unfolded point, from which a subset of local vertex combinations can lead to a specific configuration [41].

Multistability enhances the utility of metamaterials by enabling state maintenance without continuous energy input [29, 34, 35, 38, 42, 43, 44, 45]. When applied to metamaterials with writable deformation encodings, this becomes especially useful. Creating networks of bistable components with states separated by low‐energy barriers, a structure can effectively transition between many stable encodings, each corresponding to a specified shape transformation [46, 47]. Emerging inverse design approaches aim to map target geometries to configurations of multistable elements, highlighting both the promise and difficulty of navigating large combinatorial state spaces [48]. To be useful, reprogrammable multistable metamaterials need large configuration spaces, the ability to lock state, and a method of navigating the configuration space of the materials.

While previous studies have used spatially varying structural elements at distinct array positions, these approaches primarily optimize static macroscopic properties prior to fabrication. For example, tailoring the geometric architecture of microlattices has been shown to govern overarching 3D buckling mechanisms and failure modes [46]. Automated optimization algorithms have likewise been used to strategically arrange discrete unit cells and defects to maximize static strain energy density [47] or to achieve bulk isotropic and auxetic properties from non‐auxetic base components [48]. Our work advances this paradigm by moving from static optimization to dynamic, post‐fabrication programmability: rather than permanently locking the array heterogeneity during design and fabrication, TCMs couple bistable elements within a periodic array so that the localized structural state at any position can be reversibly rewritten. This decouples the shape‐programming force from the holding force to enable low‐energy navigation of a complex configuration space.

**FIGURE 1:**
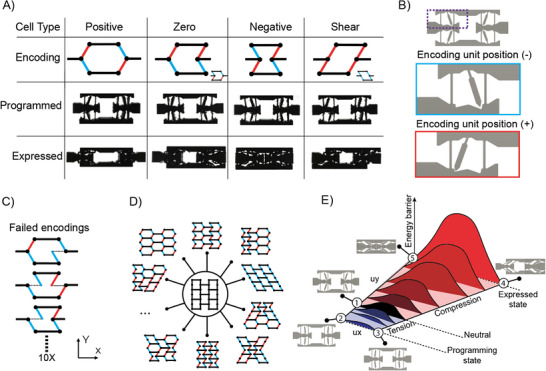
Reprogramming TCM State. (A) Each cell encoding corresponds to a different combination of BEU states. Under global compression, each cell type expresses either positive, negative, or zero Poisson's ratio or shear behavior. (B) The Bistable Encoding Unit (BEU) mechanically constrains the direction in which each joint buckles. We mechanically toggle the BEUs between two bistable positions. (C) 10 of the 16 potential trajectories for a TCM unit cell result in mechanical frustration and failure. (D) A single TCM lattice can express many different trajectories, all accessible from the central programming state. (E) We can navigate between trajectories by overcoming some strain energy barrier. Cell compression leads to larger energy barriers while cell tension reduces the required transition energy. We show a standard programming procedure with states 1 through 4. State 4 to 5 demonstrates disturbance of a stable expressed state.

Built on these concepts, we have developed Transition‐Controlled Metamaterials (TCMs) that can generate complex surface profiles within defined geometric limits and are rewritable after fabrication to encode desired shape expressions. These materials have a single programming state where low force (∼0.5 N) inputs can adjust the preferred soft mode of deformation [24] to express highly stable (>20 N disturbance force) shapes under global loading. We alter the global response of TCMs by physically constraining the buckling direction of each interior joint based on the state of “Bistable Encoding Units” (BEUs) (Figure 1A) at each cell joint in the repeating lattice.

We characterize 3D‐printed TCMs that can be actively reprogrammed using a custom encoding machine. We define required geometric conditions for predictable transitions, create an inverse method for shape matching, display 2D information, and make fully developable 3D surfaces. Our results demonstrate how the navigation of a mechanical metamaterial's state space can produce writable and stable constructs with complicated shape reconfiguration.

## 2 Results

### 2.1 Design and Reconfiguration

Mechanical metamaterials can demonstrate unusual properties based on their architected periodic structure. Like many metamaterials, Transition Controlled Metamaterials (TCMs) are constructed as a tiling of flexible unit cells made of thick segments (beams) and thin segments (flexures). Under a global compression, these structures flex based on a preferred soft mode of deformation [24] determined by the arrangement of their members. To approximate a structure's deformation, we treat the beams as semi‐rigid members and the flexures as revolute joints. Each unit cell contains four Binary Encoding Units (BEUs) (Figure 1B) that physically constrain the movement of each joint, encoding a soft mode of deformation that we define as either positive (+) or negative (‐). A BEU consists of an angled beam connected on one end to a flexure. In its initial fabricated state, the beam acts as a support, allowing the cell joints to bend one direction but not the other. We can physically displace the tip of the beam from its initial supporting position to reverse the direction of support, which biases the joint to bend in the opposite direction. A bulge between the two BEU positions forces the beam to bend and then rebound, creating a bistable system (Figure 1B). By pushing the BEU back and forth between these two bistable states, we can effectively switch the cell's soft mode of deformation and hence program its final expressed form. We follow a three‐step path to reprogram and then express a TCM's shape (Figure 1E). First, we apply tension the structure in the y direction, reducing the energy barriers between the BEU states. Second, we mechanically switch the BEU to alter the encoding. Third, we apply a global compression in the y direction to guide the system along the selected path to reach an expressed state (Figure 1A). The 4 BEUs in a single cell can be collectively configured into 16 different combinations. Of these, 10 combinations cause competition between joints with no soft mode of deformation, resulting in geometric frustration [15, 49] (Figure 1C). The remaining 6 BEU combinations correspond to one of four different cell states. We characterize cell states by positive, negative, and zero Poisson's ratios as well as a shearing state (Figure 1A). When configuring several connected cells in a TCM (Figure 1D), special care must be taken to ensure that all cells have a valid BEU combination. To achieve this, the shearing cell acts to bridge regions with unlike positive or negative Poisson's ratios. If no shearing components are included, attempting to alternate cell types between adjacent horizontal rows will result in geometric frustration. The shear cell enables spatially varying shape changes throughout multiple connected cells, including asymmetry across rows. This functionality makes it a key component to creating detailed and asymmetric profiles.

**FIGURE 2:**
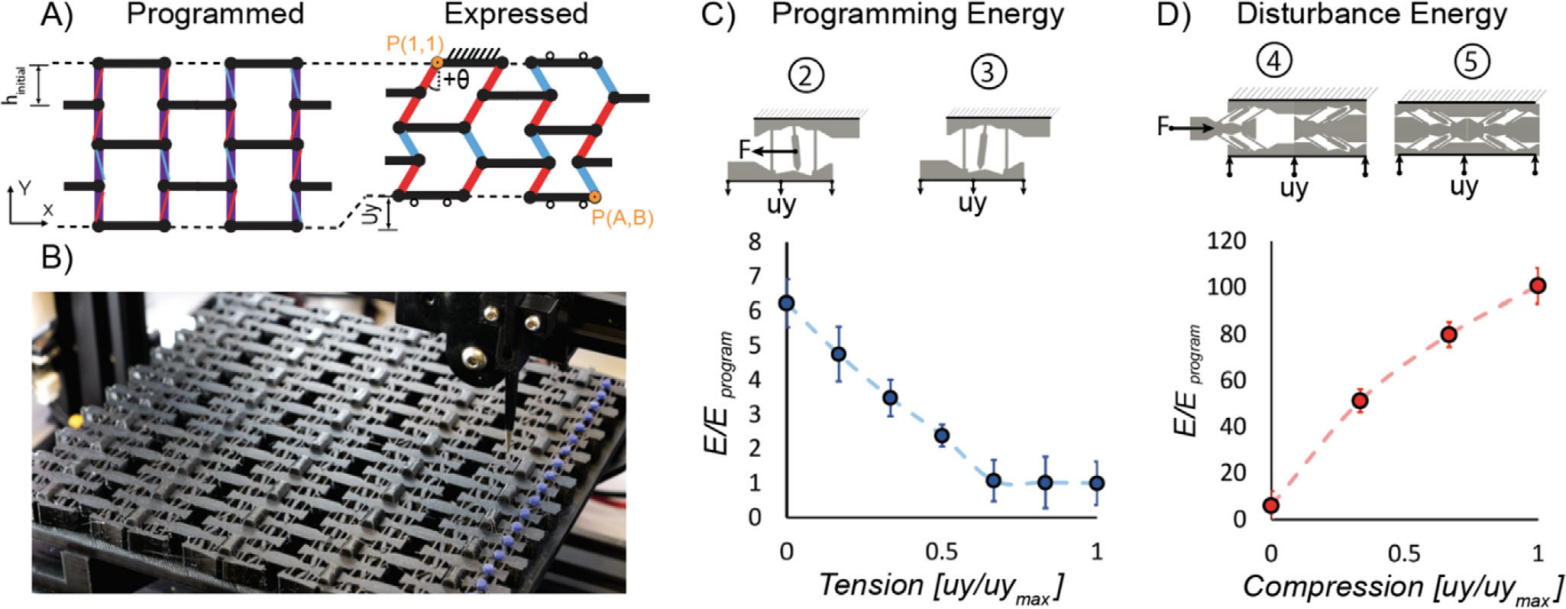
Encoding Escape Energy. (A) A programmed encoding determines the final expressed state at all defined points *P*(*a*, *b*). (B) We use a specially adapted machine to automatically reprogram TCM sheets. (C) We program the cell by nudging the BUE beams between states. The energy required to program the cell becomes smaller with extension. (D) As the cell compresses along an encoded deformation mode, we test the energy required to deviate from the given encoding. The greater the compression, the more energy it takes to switch encodings.

To reprogram TCMs, we created a machine to automatically adjust each BEU in the lattice (Figure 2B). A custom mount holds a TCM sheet in place and tensions each cell to enable low‐force reprogramming. As part of our shape‐matching pipeline, we write machine G‐code based on the desired encoding of the TCM. A motor‐driven gantry then moves an end effector across each cell, toggling the position of each BEU to its respective position (Video S1). This programming machine is an adapted fused‐deposition‐modeling 3D printer: the TCM sheet mounts on a custom peg fixture that holds and stretches each cell in the y‐direction to lower the programming force, while a gantry‐driven metal end‐effector steps to the center of each BEU and toggles its state, reconfiguring the entire lattice before global compression (Figure 2B).

To control the collective transformation of a many‐celled structure, we designed TCMs to ensure collinearity of horizontal beams at the final expressed state. As the TCM reaches a maximum compression (*uy_max_*), each cell's geometry mechanically constricts the position of linkages in the final expressed state, holding cross beams in a horizontal position (Figure 1A). To satisfy our shape matching procedure, non‐uniformity throughout the deformation is permissible given we enforce collinearity at the final expressed state. We constrain boundary conditions so that no out‐of‐place deformation occurs by housing the 2D TCM structures between two flat sheets. Connections at the top and bottom edges slide freely throughout compression to enable boundary adjustments in the x direction and resulting shape changes.

From the structure's neutral state, the TCM can be either tensioned or compressed. With tension, compliance in the beams and joints allows the BEUs to extend, reducing friction between the BUE support beam and the opposing surface, hence lowering the energy barrier between states. As the structure continues to extend (Figure 1E, state 1 to 2), the programming energy drops until the elasticity of the BEU's joint acts as the dominant force and the BEU becomes monostable (Figure 2C). At this point, the elastic energy of the joint becomes the threshold for the minimum energy required to reprogram the system. While this paper only considers a single design, adjusting the width of the flexures or changing the TCM base material will directly affect the required reprogramming energy.

As the TCM compresses (Figure 2C, state 3 to 4), the energy required to disturb the programmed encoding increases (Figure 2D, state 4 to 5). Through experimentation, we observe a two order of magnitude (>100 ×) increase in the energy required to adjust the encoding in the expressed states compared to the programming state. By decoupling the programming force from the holding force, we are able use relatively low (∼0.5 N) force actuation to encode the material and achieve much higher (>20 N) holding forces after compressing the material. Additional work could explore how spatially varying cell type and compression level affect not only shape but physical properties of the material as well. We distinguish three separate processes when operating a TCM: programming an individual BEU at low force (∼0.5 N), globally compressing the lattice to express the shape, and disturbing or holding the expressed state (>20 N). The force required for global compression depends on the programmed configuration. Single‐cell compression tests show that reaching full compression requires about 12.2 N for an auxetic cell, 10.5 N for a zero‐Poisson‐ratio cell, 9.0 N for a positive‐Poisson‐ratio cell, and 7.74 N for a shearing cell. This corresponds to effective stiffnesses of roughly 2.038, 1.507, 1.746, and 1.289 N/mm, respectively. By modeling the lattice as a spring network, we find that the lattice's effective stiffness depends primarily on the base stiffness and its aspect ratio (N/M) (Supporting Information). Because each state has a different effective stiffness, the exact programming state would vary but be bounded by a pure auxetic and shearing state stiffness times the aspect ratio.

### 2.2 Sparsity of Valid Encodings

From the programming state, we can easily transition between different potential trajectories. For the TCM to compress along a single soft mode of deformation, all BEU encodings must collectively transform together to avoid competition and mechanical frustration. To approximate the expression of the collective system, we treat TCM beams as rigid members and flexures as revolute joints. Horizontal crossbars alternate to connect every other grid point to the adjacent grid point, adding geometric constraints to the system. As the structure compresses by a given displacement (*uy*), the angle of the rotation for each vertical beam can be approximated as ($\theta =\cos^{-1}\left(\;\frac{h_{initial}-uy}{h_{initial}}\;\right))$. Based on these assumptions, the x position of each joint in a TCM with an array of (*A* × *B*) BEUs can be expressed as

$$P{\left(a,b\right)}_{x}=P{\left(a,0\right)}_{x}+\sum_{i=0}^{b}h_{initial}*\cos(\theta )*EV\left[a,i\right]$$

To test whether a given encoding leads to a valid expression, we can evaluate the expressed state of the system at *uy_max_* and check to see if the lengths of all rigid beams have been maintained (Supporting Information).

To derive an expression for the total number of valid configurations, we used a brute force method (Supporting Information) to generate and test every candidate combination of an *A* × *B* BEU array up to A*B < 50 and *A*, *B* < 16. As the number of lattice linkages grows, the number of total possible combinations (2^A*B^) increases very rapidly, while number of combinations leading to a valid expression ($2^{k_{1}AB+k_{2}\left(A+B\right)+k_{3}}$, *k*_1_ = 0.2989,  *k*_2_ = 0.6924, *k*_3_ = −1.3831) increases much more slowly (Supporting Information). Following this trend, the number of valid states grows quickly but the probability of randomly selecting a valid configuration from the transition state rapidly approaches zero as the size of the tiling grows. For a 10 × 10 array, there are approximately 5.62 · 10^12^ valid states but 1.2677 · 10^30^possible combinations. Hence, the probability of selecting a valid state at random is only 4.433 · 10^−16^percent. This sparsity of valid encodings for a TCM lattice makes manual selection of possible states extremely difficult and necessitates an inverse strategy to generate valid encodings.

### 2.3 Profile Matching

Our profile matching pipeline consists of three main categories, initial shape processing, encoding generation, and physical expression. We offer detailed descriptions of encapsulated sub‐steps in the Supporting Information.

Our shape processing function accepts two input types, hand drawn profiles or single‐contour SVG files. Limitations exist for the profiles that we can successfully approximate, and we must first adjust the input contours to account for these (Supporting Information). To process the files, we segment the shape into two independent profiles, a left‐side and a right‐side profile, with a given distance separating the two. We represent these profiles as functions, in which a single y value corresponds to a single x value, by stepping along the profile from top to bottom and removing all overlapping segments of the profile (Supporting Information).

Once we have three significant components, a left‐side profile, a right‐side profile, and a distance between the two profiles, we match a valid encoding to approximate the shape. Given a TCM with a set size and a predetermined max compression, a perfect shape match does not always exist. As a result, we must make trade‐offs for which traits we preserve and disregard in a complicated shape. With our shape matching algorithm, we prioritize first the right‐side profile, then the left‐side profile, and lastly the distance between them. Alternative constructions could refine the shape matching algorithms to balance different traits for different applications. We prioritize the two side profiles because, unlike prior structures that generally produce symmetric or single‐surface profiles, TCMs express two independent complementary profiles bridged by an internal structure, and we anticipate these side profiles to be the functional surfaces. Multiple valid encodings can produce a similar target shape; our aim here is a tractable O(N) heuristic that reliably yields feasible, high‐quality encodings rather than a globally optimal solution, and we leave a formal study of optimality and its objective criteria to future work.

The first step in the encoding generation is to approximate the left‐side and right‐side profiles as a combination of discrete (+) and (‐) sloped line segments with a slope of (θ_*max*_). For a lattice with (*A* × *B*) BUEs, the number of discrete segments is equal to the number of rows (B). Since the state of each upper segment effects the position of all lower points, this becomes a combinatorial problem with 2^*B*^ different options. To solve this, we first find a sub‐optimal solution by naively generating parent combinations using a greedy algorithm. Then we refine our solution by searching through superior children (lowest root‐sum squared error) with a best‐first binary search tree to find a quasi‐optimal profile encoding (Supporting Information).

To create a valid encoding for the entire structure, we start with the chosen right‐side encoding, then move leftward column by column until we attain the prescribed left‐side profile. As we step from one column to the next, each presents an additional 2^*B*^ possible combinations, making it an intractable search space. By leveraging several geometric constraints, we can reduce this search space and instead effectively match the shape with an O(N) complexity greedy algorithm (Supporting Information). We generate these constraints by evaluating the options for valid unit cells (Figure 1A). Given a unit cell, if the right‐side BEUs are set with predetermined encoding, the left‐side BEUs have only 4 potential options, (−1, −1), (+1, +1), (−1, +1), and (+1, −1). If the right‐side is either (+1, +1) or (−1, −1), then the encoding must be a shear cell and the left‐side encoding must equal the right‐side encoding. When performing our full encoding generation, we can first iterate through each pair of BEUs and assign all shear cells in the following column based on the state of the current column. For the remaining BEU pairs in the following column, we can then assign only two options, (+1, −1) or (−1, +1). To do this, we step down each column and compare x‐values for each of the two options (Supporting Information). We select a favorable BEU pair based on which more closely matches our target left‐side profile. We iterate through all columns to generate an array of encoding values (EV) populated with either +1s or −1s. Although not universally optimal, this algorithm generates valid encodings that closely match aspects of the overall shape. We can simulate the transformation of an overall lattice by evaluating the position of all points at discrete compression values 0: *uy_max_* (Supporting Information) (Video S2).

### 2.4 2D and 3D Physical Expression

**FIGURE 3:**
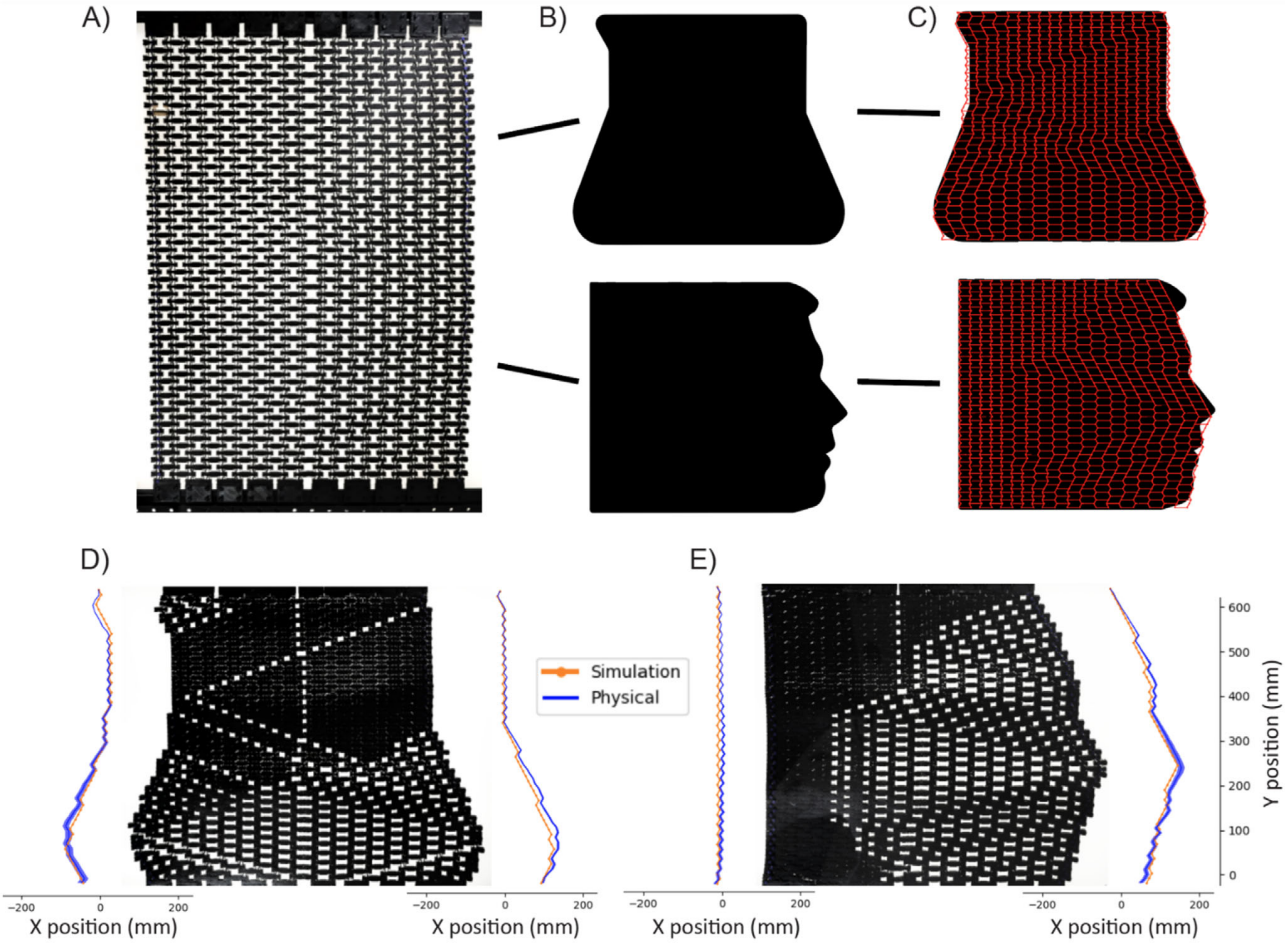
Complex shape generation. (A) Neutral lattice position with a programmed encoding. (B) Two desired shapes which we fed into our profile matching pipeline. (C) Simulated expression of the resulting TCM configurations. (D) Physical expression of a beaker shape with comparisons of the expected and real profiles. (E) Physical expression of a face shape with comparisons of the expected and real profiles.

We 3D printed TCMs to create reprogrammable 2D profiles and 3D surfaces. To match complicated profiles (face and beaker, Figure 3B) (Video S3), we printed six 10 × 20 unit TCM sheets, which we connected to form one larger 20 × 60 sheet (Figure 3A). We generated an encoding for each profile (Figure 3C) and programmed each sheet using our custom machine (Supporting Information) (Figure 1E). To express each desired shape, we used a linear stepper motor to apply a global compression of *uy_max_* = 165*mm* to the TCM. We attached markers to each joint on the TCM's left‐side profile and the right‐side profile to perform optical tracking (Supporting Information) and compared our physical results against our simulation results for each profile expression. In the fully expressed state, physical and simulated profiles matched with an average root mean square (RMS) strain error ε_*x*_ = 0.0105 ± 0.0044 . These experiments demonstrate general agreement between our simulated and physical models in the expressed state. Uneven deformation in the flexible structure likely accounts for much of this observed error, and future work could explore the uneven behavior of TCMs throughout their entire deformation. Additionally, it is worth noting that these experiments do not reflect the overall ability of the structures shape matching capabilities, which varies greatly depending on the target profile. To separately quantify how closely the simulated expression reproduces the intended target, we added a normalized root‐mean‐square (RMS) error between the target and simulated profiles (Supplementary Materials). Across two representative target shapes, the left‐ and right‐side errors were 0.48% and 1.95%, and 0.65% and 0.68% of the shape width, and varied with the achievable local slope and the number of cells.

**FIGURE 4:**
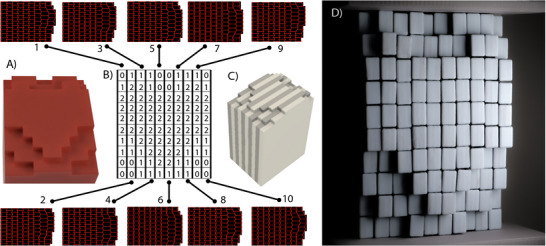
3D Surface expression. Stacking multiple TCM sheets creates reprogrammable 3D height maps. (A) Target shape which was sliced and fed into the shape matching pipeline. (B) Simulated expression of each layer and its associated height value. (C) Ten individual layers stack to create the developable surface. (D) Physical expression of the 3D model.

We created reprogrammable 3D structures with fully developable surfaces by stacking several layers of TCMs. Our 3D system (Figure 4) consists of ten 10 × 20 unit TCM sheets stacked parallel to one another. To approximate a 3D profile, we started by generating a computer‐aided design (CAD) model of our desired shape. We split this design into several layers and saved the resulting contours as SVG files. Then, we processed each 2D shape with our shape matching protocol and programmed each layer with our custom machine. We constrained the outer surfaces of the structure with flat, sliding contacts and globally compressed the structure to express the desired shape. For this demonstration, we additionally constrained the structure to express a flat‐backed profile and specifically focused on the expressed heart figure on the front of the TCM array (Figure 4C and Video S4). To compare our physical and simulated shape expressions, we once again attached markers to the edges of each layer and analyzed deformation using optical tracking.

We demonstrate successful simulation and physical expression of TCM's 2D and 3D shape matching capabilities, however, several constraints limit the functionality of these structures. First, the resolution of profile representation is inherently limited by the density of TCM cells in the lattice. While these structure's behavior is purely driven by geometry, enabling potential uses at many size scales, small features will be needed for high resolution reprogrammable displays. Next, the shape of potential approximated profiles is limited by the angle (θ_*max*_) achieved at maximum compression. Adjacent cells in a TCM's profile cannot vary more than one positive or negative unit step at a time, meaning that it is difficult to approximate flat lines or overhangs. In our physical model, (θ_*max*_) = 28 *deg* based on our geometric design.

## 3 Discussion

This work offers an effective strategy for programming complicated and re‐writable shape changes using Transition‐Controlled Metamaterials (TCMs). As demonstrated, specific combinations of programmable bistable linkages may be used to navigate between deformation pathways. We access a single programming state by globally tensioning TCMs, from which point we may access all other valid configurations with low energy actuation. A viable shape matching pipeline closely represents complex profiles within defined geometric limits and enables automatic encoding of deformation using only small mechanical nudges to program the system. Experimental testing demonstrated close agreement between simulated and physically expressed profiles for both 2D and 3D structures. Because this framework is based on rigid‐body kinematics aimed at rapidly navigating a highly degenerate space of geometric combinations, it does not compute internal stress distributions within the members; instead we characterized the relevant energy barriers and forces experimentally. This strategy decouples programming force from actuation, creating opportunities for increased scalability and improved resolution. In addition, it supports stable mechanical memory and requires increased forces (>20 N) to disturb a state once expressed. This concept is scale‐independent, allowing the strategy to work at the scale of MEMS devices up to architectural surfaces. TCMs offer opportunities to fundamentally change human‐computer interaction through object simulation, communication of visual and tactile information, user augmentation, and extended reusability [1, 6]. Reprogrammable structures have utility in digitally adjustable tooling and jigs, variable friction materials, tuneable acoustic surfaces, and robotic grippers, locomotion, and camouflage [17]. The principal advantages of TCMs are their mechanical memory, the low force required for programming, the decoupling of programming force from holding force, and the ability to maintain an expressed shape without continuous energy input. These advantages are accompanied by limitations: in its current serial implementation the reprogramming process is relatively slow, programming requires an external encoding machine, the spatial resolution is bounded by the cell size, and the achievable profiles are constrained by the lattice geometry. Because TCMs are built from flexures, application‐specific characterization of fatigue, hysteresis, wear, manufacturing variability, and failure modes over many programming cycles is an important direction for future work.

## 4 Methods

### 4.1 Conditions for Valid Lattice Encoding

To effectively control collective transformations in a many‐celled TCM, we tuned our design to ensure collinearity of horizontal beams, connected asymmetrical regions with the shear state, and matched shape with a 9‐step process (Supporting Information S6–S8). We enforced boundary conditions so that no out‐of‐place deformation occurred and connections at the top and bottom edges slid freely to adjust for shape changes. To maintain collinearity in the expressed state, we specifically designed each cell to mechanically constrict the position of linkages in the final expressed state (Figure 1B). For our shape matching procedure, non‐uniformity throughout the trajectory is permissible as long as we enforce collinearity at the final expressed state. For a TCM with *A* × *B* BEUs, we developed criteria to test whether a given joint combination results in a valid state. Every possible valid state of the TCM makes up a finite subset within the total 2^A*B^ possible combinations of array values. Horizontal crossbars with a length of *w* alternate to connect every other grid point to the adjacent grid point, adding geometric constraints to the system. First, we calculate the x‐position of the top points in each of the TCM columns, *P*(1, 1: *B*). This can be done by summing all horizontal beam lengths plus the offsets created by the vertical beams (*k*) (Supporting Information S3A). For a vertical beam length (*h*) and a y‐displacement (*uy*), we express the beam angle as $\theta =\cos^{-1}\left(\;\frac{h-uy}{h}\;\right)$, and the resulting offset created by the beam as *k* = h*sin (θ). Given an array of positive and negative encoded values [EV], we then calculate the position of each top point.

$$P{\left(1,b\right)}_{x}=b*w+\sum_{i=2,i\;even}^{b-1}\left(EV\left[1,i\right]*k\right)-\sum_{i=3,i\;odd}^{b}\left(EV\left[1,i\right]*k\right)$$

With these starting values, we express the x‐position of every point in a TCM.

$$P{\left(a,b\right)}_{x}=P{\left(1,b\right)}_{x}+\sum_{i=a}^{a}h_{initial}*\cos(\theta )*EV\left[i,b\right]$$

After calculating the x‐position of all points, we iterate through the TCM and check that all horizontal beams maintain their original length in the final expressed position. We consider a TCM configuration to be valid given the conditions.

$$\begin{array}{cc} & (P{\left(a_{even}+1,b_{even}\right)}_{x}-P{\left(a_{even},b_{even}\right)}_{x}\\ & \;=w)\;\forall \;\left(1\leq a\leq A,1\leq b\leq B\right)\;\mathrm{And}\;\\ & \;\left(P{\left(a_{odd}+1,b_{odd}\right)}_{x}-P{\left(a_{odd},b_{odd}\right)}_{x}=w\right)\;\forall \;\left(1\leq a\leq A,1\leq b\leq B\right) \end{array}$$

### 4.2 Size of Valid Combination Space

Our simulations are purely kinematic and geometric: rather than using finite element analysis or solving dynamic equations of motion, we treat the thick beams as semi‐rigid members, and the thin flexures as revolute joints and directly compute the spatial positions of all expressed points. By brute force computation, we calculated the position of all expressed points and tested whether link lengths are maintained for all horizontal cross bars. We iterated through all 2^A*B^ possible BEU combinations to measure the number of successful outcomes for a given TCM. To derive an expression for the total number of valid leaf nodes, we generated and tested every candidate combination of an *A* × *B* linkage array up to A*B < 50 and *A*, *B* < 16. This data created a symmetric matrix with 25 total points (Supporting Information S6). Of these combinations, we selected 17 points to act as fit data, and 8 points to act as validation data. By taking the *log*_2_ of the fit data for valid configurations, we were able to generate best fit lines for *A* = 2, 4, 6, 8 as B increased, with *R* ^2^ >.99998. The slope and intercepts of these four lines also fit a linear relationship as the A value increased, such that *R* ^2^ >.99999. This logarithmic relationship and the two linear equations combined to create a single general equation to describe the valid combination space as A and B varied. The number of total valid combinations = $2^{k_{1}AB+k_{2}\left(A+B\right)+k_{3}}$ with the three constants, *k*_1_ = 0.2989,  *k*_2_ = 0.6924, *k*_3_ = −1.3831, obtained through the linear fits. We tested this general equation using our validation data and achieved *error* <2.1% for all points (Supporting Information S6). As the number of lattice linkages grows, the number of total possible combinations (2^*A* × *B*^) increases very rapidly, while number of combinations leading to a valid trajectory ($2^{k_{1}AB+k_{2}\left(A+B\right)+k_{3}}$) increases much more slowly. Following this trend, the number of valid states grows quickly but the probability of randomly selecting a valid configuration from the transition state rapidly approaches zero as the size of the tiling grows. For a 10 × 10 array, there are approximately 5.62*E*12 valid states but 1.2677*E*30 possible combinations. Hence, the probability of selecting a valid state at random is only 4.433*E* − 16 percent. For such large lattices, the chances of randomly selecting a valid trajectory quickly approaches zero, necessitating an inverse strategy to generate a valid encoding.

### 4.3 Programming Energy

To test the force and energy required to program a cell's trajectory at a predefined level of extension, we experimentally evaluated 3D‐printed TPU unit‐cell samples using an Instron Universal Testing System. To perform these tests, we physically constrained the horizontal beams of the unit cell with an adjustable mechanism that pulled the cell beams to a specified width. Next, we used a small metal beam to pull or push a single BEU beam forwards and backwards across the mechanical energy barrier of the joint. We tested seven different tension levels (*uy_max_*/(1,2, 3, 4, 5, 6, 7)) with their relation to ‘maximum tension’, which we describe as the point at which the center beam became free to move back and forth with no mechanical interference (*uy_max_* = 3.5*mm*). We tested 5 samples per point and plotted their average force vs deformation in Supporting Information S5D. Here, thickness of the line shows the standard deviation between samples. We calculated the total programming energy as the sum of all forces (*F*) over the deformation in the x direction (*ux*) at each measured point (*p*), such that $E_{Program}=\sum_{p=0}^{p_{Program}}\left(ux(p+1)-ux(p)\right)*F\left(p\right)$. From these experiments, we observed an 84% reduction of required programming energy from the neutral (7.88 *mJ*) to the fully extended state (1.265 *mJ*).

### 4.4 Disturbance Energy

To test the force and energy required to disturb a cell's trajectory at predefined levels of compression, we experimentally evaluated 3D‐printed TPU unit‐cell samples using an Instron Universal Testing System. To perform these tests, we physically constrained the horizontal beams of the unit cell with 3D‐printed PLA jigs. Next, we fixed each sample in one of the three possible programmed BEU configurations (inwards trajectory, outwards trajectory, and shear trajectory). We tested four different compression levels with their relation to maximum compression (*uy_max_*/(1,2, 3, 4)) with *uy_max_* = 5.5*mm*. At each compression level, we performed the tests by applying a deformation to the center of the cell's unsupported beam in the direction opposite the direction of the initial programmed trajectory (Figure 2). We deformed the cell until we effectively disturbed the state of the programmed trajectory. We defined this as the point as the moment when the BUE's of the cell switched position, resulting in a change in the programmed trajectory of the cell. We tested 5 samples at each of the positions and plotted their average force vs deformation in Supporting Information S5. Here, thickness of the line shows the standard deviation between samples. We calculated the total disturbance energy as the sum of all forces (*F*) over the deformation in the x direction (*ux*) at each measured point (*p*), such that $E_{Disturb}=\sum_{p=0}^{p_{Disturb}}\left(ux(p+1)-ux(p)\right)*F\left(p\right)$. For all configurations, we see large (>7*x*) increases in required disturbance energy from the uncompressed to the fully compressed states.

### 4.5 Profile Matching Pipeline: Shape processing

Our shape processing function accepts two input types, hand‐drawn profiles or single‐contour SVG files. While we can accept any contour, several limitations exist for the profiles that can be successfully approximate with our TCMs, so we must perform several pre‐processing steps to fit the desired shape criteria. First, we must split a shape into two side profiles, left and right. We start by converting the.svg file into many cartesian (x,y) points and iterate through each point, recording the maximum and minimum y values. Next, we sort points within the top 2% of y‐value subset and select the leftmost and rightmost points as the start of our left and right contours. We step along each contour until we reach the bottom 2% y‐values in the image to select our complete left and right profiles. Next, we need to represent each profile as a function, with a single y point returning a single x point. This means that we must remove any overhangs or horizontal lines in the profile. We iterate through points from the top to the bottom on the left and right profiles. For any point (P) with *P*(*i* + 1)_*y*_ < *P*(*i*)_*y*_ we replace *P*(*i* + 1)_*y*_ = *P*(*i*)_*y*_ −.001. This removes the overhang and horizontal lines, replacing them with a slightly slanted line. Finally, we interpolate between points so that any input y value returns a valid x value. The resulting functions generate single values *f*(*y*)_*goal*_ that we can then feed into the shape matching function.

### 4.6 Profile Matching Pipeline: Generating Encoding

Once we have valid functions to represent both the left and the right sides of the contour, we then create a valid encoding that can be programmed into the TCM structure to create a physical expression matching the desired shape. First, we generate an encoding for both the right and the left‐side profiles. For a TCM lattice with BUE row count = A, we must approximate each profile with A‐1 connected line segments with defined positive or negative slopes ± θ (Supporting Information S3). For a single column of BEU encoding values (EV[1:A,1]), we can represent the cartesian coordinates of each point (P(a,1), in the profile as $P{\left(a,1\right)}_{x}=\sum_{i=1}^{a}h_{initial}*\cos(\theta )*EV\left[i,1\right]$ and $P{\left(a,1\right)}_{y}=h_{initial}-\sum_{i=1}^{a}h_{initial}*\sin(\theta )$. From our shape processing procedure, we can represent the ideal profile with the function, *x_goal_* = *f_match_* (*y*). We calculate the error between the goal and the approximated profile x values at each point as *error_x_* = |*P*(*a*)_*x*_ −  *f_match_* (*P*(*a*)_*y*_)|. To generate encoding values (EV) for each profile, we first use a greedy algorithm to create an initial approximation of the shape. We step through each row (1: *A*) and append either +1 or ‐1 to minimize *error_x_* at each point. This approach creates a sufficient match for relatively flat profiles but can result in large errors when the target shape contains dramatic curves. Since upstream values in the chain of connected beams effects all downstream values, we must consider all 2^*B*^ combinations to discover the optimal solution. To reduce profile approximation error, we first use our greedy algorithm to generate profiles starting from the top and bottom of the profiles (1: *A*,  *A*: 1). Then, we search through all encoding values (EV) combinations that disagree between the two initial samples using a best‐first binary search tree. As we traverse the tree, if the root sum squared (RSS) error becomes greater than the RSS error from the original greedy algorithm, then we can prune the current branch and reduce the search space. To keep computation times low, we stop our search after a set number of recursions (1,000). While this algorithm does not guarantee an optimal solution, it does generate a qualitatively similar profile for all tested shapes and reaches an optimal solution for TCMs with low cell counts (Figure 3).

Once we have generated satisfactory encoding for the left‐profile and the right‐profile, we then fill in the encoding values for all remaining cells in the array. To generate a valid encoding for the entire structure, we step from the initial right‐side encoding and individually fill columns until we reach the desired left‐side profile. We detail this algorithm in the body of the paper and provide additional details in Supporting Information S7 step 6.

### 4.7 Profile Matching Pipeline: Programming and Expression

To physically program and express the desired shape encoding, we use a custom machine to adjust all binary encoding units (BEUs) based on generated G‐code (Video S1). We start by mounting the TCM sheet onto a custom peg fixture that holds and stretches each cell in the y‐direction to reduce programming force and ensure accurate positioning. With a desired array of encoding values [EV] and known TCM dimensions, we program an adapted FDM 3D printer to move a metal end‐effector to the center of each BUE and sequentially program the TCM. The machine performs a set of programming steps based on the associated EV values at each BEU. If *EV*[*a*, *b*] = −1, then the end effector steps right in the x‐direction, down in the z‐direction, left in the x‐direction to switch the BEU state, back up in the z‐direction, and finally returns to center. If *EV*[*a*, *b*] = +1 then the machine performs a similar series of movements but mirrored vertically about the center of the BUE, moving left for the first step instead of right. The end effector iterates through each of the columns, adjusting every BEU individually to fully reconfigure the structure encoding. While this design successfully demonstrated the concept and automated the shape reprogramming process, future iterations could achieve higher programming speeds by parallelizing the end effectors to adjust many cells at once. To express the shape change of the TCMs, we connected several individual sheets to make a larger TCM array. We connected these arrays to sliding contacts at the top and bottom of the array and compressed them using a Nema 23 stepper motor connected to a linear screw actuator.

### 4.8 2D and 3D Structure Fabrication

To fabricate reprogrammable 2D profiles and 3D surfaces, we 3D printed TCMs from a 90A durometer Thermoplastic Polyurethane (TPU). The base unit of each TCM is comprised of a single BUE with a width of 17.56 mm and a height of 8.05 mm. We tiled the BEU's with the ppm wallpaper group pattern (*2222 orbifold notation) in a 10 x 20 array to make the full lattice. To simplify fabrication, we printed the TCMs as initially biased toward the negative Poisson's ratio state by 10 degrees to avoid interference and fusing between the BEU beam and the other components while 3D printing. We added dovetail joints at the top and the bottom of the sheets to allow easy connection between single sheets. This allowed us to combine 2x3 individual sheets together to create a larger array of TCMs. The dove tail joints also allowed us to connect the TCM lattice to 3D‐printed rollers to create sliding boundary conditions at the top and bottom. On the sides of the TCM sheets, instead of using the printed dovetail joints, we glued PLA dovetail joints to connect adjacent cells. In Figure 3, this can be seen as a slight horizontal gap at the center of the large TCM lattice in the expressed state. To create the 3D structures, we built an enclosure to house and compress multiple stacked layers of TCMs. Our system included two flat 3D‐printed PLA end pieces, one of which remained fixed in place, and the other which slid along four rectangular acrylic bars to compress the TCMs. The acrylic bars constrained the TCMs on each of the flat surfaces to resist any out‐of‐plane deformation. The two remaining sides of the TCM stack remained free to deform, allowing them to express the desired shape with the given compression.

### 4.9 Optical Tracking

Optical tracking compared the expressed results from our physical and simulated models. We compared the profiles independently on the right and left side since our shape matching algorithm did not optimize for distance between profiles but instead prioritized relative features on each side. To perform optical tracking of the physical model, we 3D printed circular markers from blue PLA plastic and glued each marker to the center of each joint. We then compressed each planar structure and photographed the expressed shape with a Panasonic Lumix GH5 digital camera. We used the opensource library OpenCV with python to perform post processing on the images. First, we filtered each image based on color to remove everything that did not have a blue hue. Next, we filter the photo to remove noise by applying a 5x5‐pixel gaussian blur followed by a sharpening kernel. We convert the image to greyscale apply a threshold to leave only the brightest regions then dilate and erode the image to remove any remaining small blobs. Then, we use OpenCV's Simple Blob Detector to mark every circle and return then coordinates of the center of each marker. We converted each image from pixels to millimetres and calculated the error between the tracked physical points and the simulated points. All error values for each point can be found in Figure S9.

## Author Contributions

J.I.L. and S.T. worked together to conceive of and implement the concepts.

## Conflicts of Interest

The authors declare the following competing interests: they have submitted a provisional patent application 63/147,001 based on this work.

## Supporting information




**Supporting File 1**: advs76850‐sup‐0001‐SuppMat.docx.

**Supporting File 2**: advs76850‐sup‐0002‐MovieS1.mp4.

**Supporting File 3**: advs76850‐sup‐0003‐MovieS2.mp4.

**Supporting File 4**: advs76850‐sup‐0004‐MovieS3.mp4.

**Supporting File 5**: advs76850‐sup‐0005‐MovieS4.mp4.

## Data Availability

The data that support the findings of this study are available from the corresponding author upon reasonable request.
