# Supplementary material for: Bistable Networks Enable Complex Shape Changes
Source: Adv Sci (Weinh). 2026 Jul 31:e76850. Online ahead of print. doi: 10.1002/advs.76850 (PMC13427233; doi:10.1002/advs.76850)
Supplement: Supplementary file 1 — Supporting File 1: advs76850‐sup‐0001‐SuppMat.docx. [file ADVS-9999-e76850-s003.docx]

**Supplementary Materials:**

**Videos**

**Video 1:** The video *SV1.mp4* shows automatic programming of Transition Controlled Metamaterials using our custom encoding machine.

**Video 2:** The video *SV2.mp4* shows profile matching and simulation using our shape matching pipeline.

**Video 3:** The video *SV3.mp4* shows physical profile matching for both the face and the beaker profiles.

**Video 4:** The video *SV4.mp4* shows physical expression of the 3D heart surface.

**Base Lattice Selection.** While we specifically tailored TCM geometry to enable predictable reprogramming, the basis of the TCM design stems from the standard honeycomb and auxetic reentrant honeycomb^1–5^ pattern (S1.B). We selected this lattice based on three intrinsic properties. First, given an unconstrained range of motion at each revolute joint, the state space of the lattice makes up a star graph ${(K}_{1,K})$, with all leaf nodes being accessible from a single central node. Second, the star graph configuration contains complex shape change enough valid states to enable complex shape change. For example, lattices such as the double arrowhead^1,6–8^ (S1.A, S2.B) or chiral structures^9^ (S2.A) support the star graph configuration but have small state spaces that are limited by geometric constraints. To ensure that the number of valid states grows rapidly as cell count increases, adjacent cells must be independently programmable. Finally, individual cells of the structure must be capable of switching between Poisson’s ratio signs. For both the reentrant honeycomb and the double arrowhead structures, the instantaneous Poisson’s ratio can be set to either a positive or a negative value based on the interior joint angle of $\theta$. This property allows the width of compressed cells to be programmatically set, enabling shape change within the lattice. As shown in figure S1, the transition between positive and negative Poisson’s ratio during a compressive trajectory occurs at maximum extension, at which point each has an angle of $\theta$ =$0$. As a result, any cell with an initial angle $\theta$ >$0$ will expand laterally as the structure is compressed, and any cell with an initial angle $\theta$ <$0$will contract laterally as the structure is compressed. As a contrasting example, the rotating squares structure^9^ (S1.C) remains auxetic throughout the entire trajectory of $\theta$. This makes the rotating square structure a poor candidate for generating varied edge profiles (S1.C).

As shown in S2.A, the 4-bar chiral lattice^1^ is one such geometry that demonstrates the desired star graph state structure. Here, the current trajectory of the structure can be defined by a combination of zero, clockwise, or counterclockwise center rotations and linkage rotations for each unit cell. As the cell count for the lattice increases, the chiral star graph has an exponentially increasing number of valid accessible leaves. However, each unit cell can only take on negative or zero Poisson’s ratios and no positive Poisson’s ratio options exist. This limits the shape changing capabilities of the lattice, requiring external shearing forces to generate horizontal deformation. Having no positive Poisson’s ratio state also limits the ability of the lattice to vary cell type in multiple directions.

The double arrowhead lattice (S2.B) also matches the star graph condition, with state configurations defined by the values of each unit cell joint angle $\theta$. For a lattice with AxB joints, there are $2^{A*B}$ potential joint combinations. However, having no shearing configuration, the number of valid configurations is greatly limited by geometric restrictions. Unit cell type can be adjusted in stripes, but like the chiral lattice, local cell changes in two directions are limited. To maintain valid physical linkage configurations in the lattice, all cells within a row must maintain a constant value of $\theta$. This means that cell type in the lattice can only be adjusted column by column, reducing the number of total valid combinations from $2^{A*B}$ to $2^{B}$. With these physical restrictions, we can adjust the effective global Poisson’s ratio of the double arrowhead lattice, but we cannot generate spatially varying Poisson’s ratios or complex profiles. It should be noted, these restrictions exist with the assumption that the lattice structure remains in the 2D plane. Out of plane deformations may open an even broader design space for deforming lattice structures with star graph representations.

**S1. Transition modes for auxetic lattice structures. A)** The double arrowhead lattice transitions from auxetic to non-auxetic. **B)** The honeycomb structure transitions from auxetic to non-auxetic. **C)** The rotating squares lattice remains auxetic throughout the full range of $\theta.$


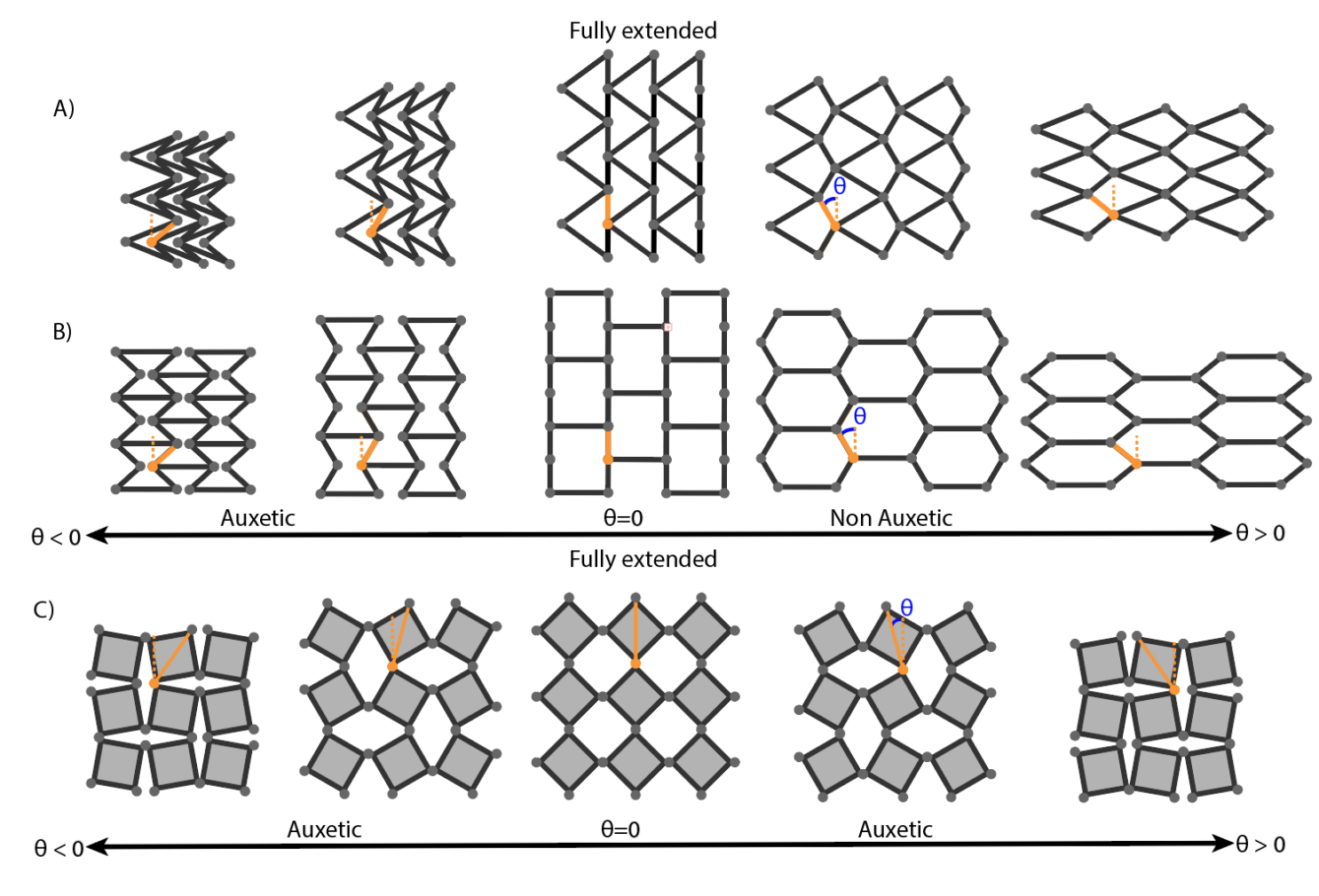


**S2. Star graph configurations for chiral and double arrowhead patterns.** **A)** A chiral lattice transitions from the central state to many different configurations with combinations of element shear and rotation. Blue denotes CW rotation, red denotes CCW rotation and grey denotes no rotation. **B)** An$A\times B$ double arrowhead lattice with A=6 and B=3 linkages can transition from the central node to $2^{B}=8$ different states. For failed combinations, dashed orange lines show discontinuities resulting in linkage deformation.


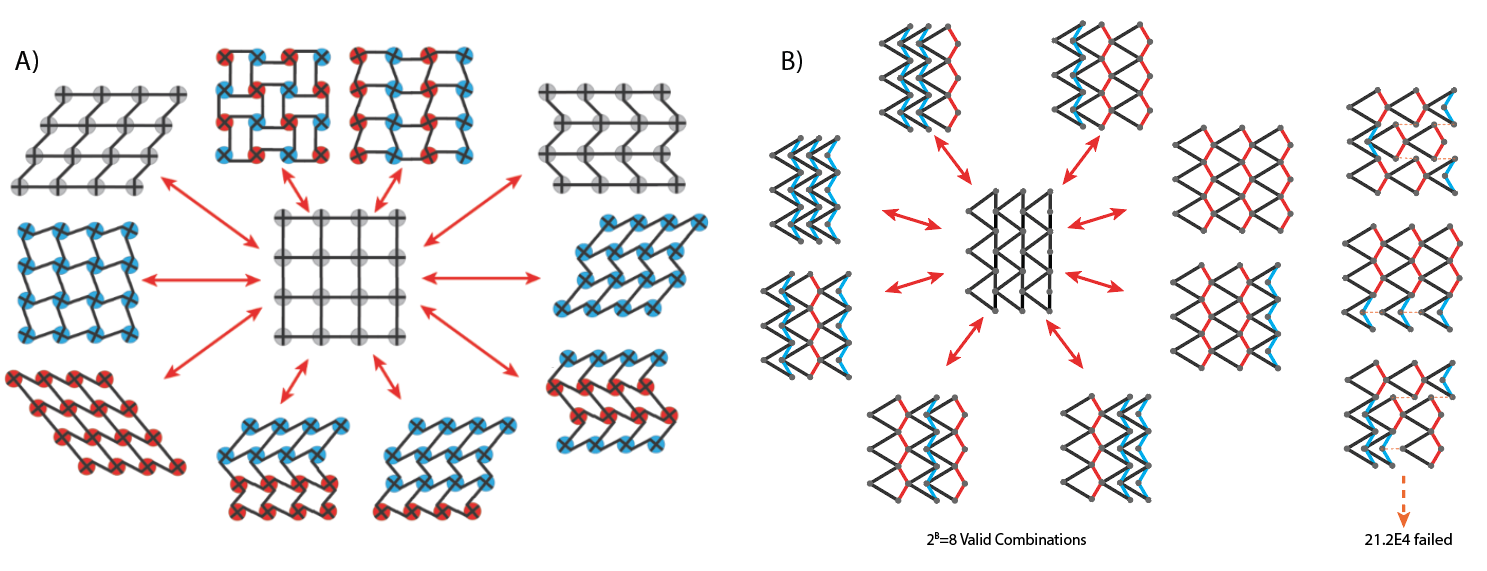


**S3. Example of Valid Structure Combination Check. A)** We encode a structure’s vertical linkage slope as either a 1 or a -1 to make up the encoding values matrix (EV). Horizontal linkages span alternating joints to define a valid configuration. **B)** Invalid link lengths define a failed configuration.


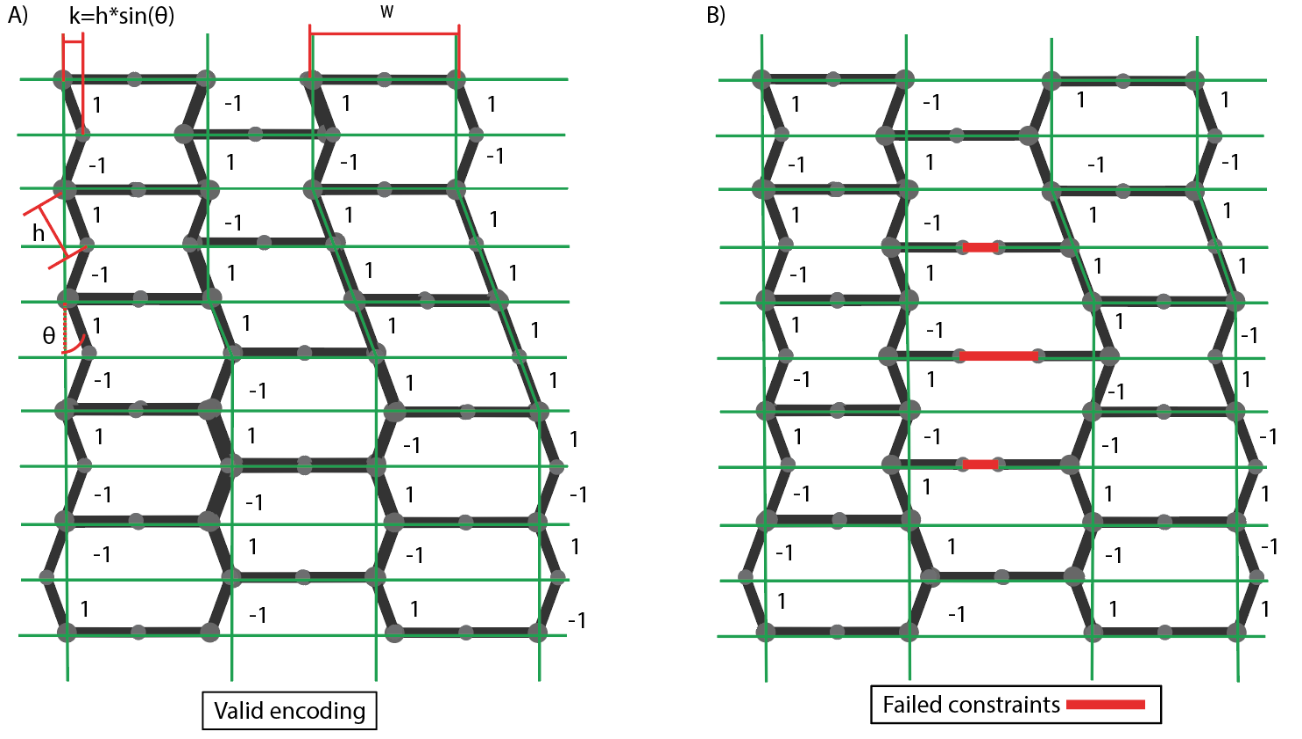


**S4.** **Combination space for total joint combinations and valid joint combinations.** As lattice cell count increases, total joint combinations expand far more rapidly than valid cell count. Tables A and B display the valid combination count and the total potential combination count in relation to $A\times B$ linkage number lattice dimensions.


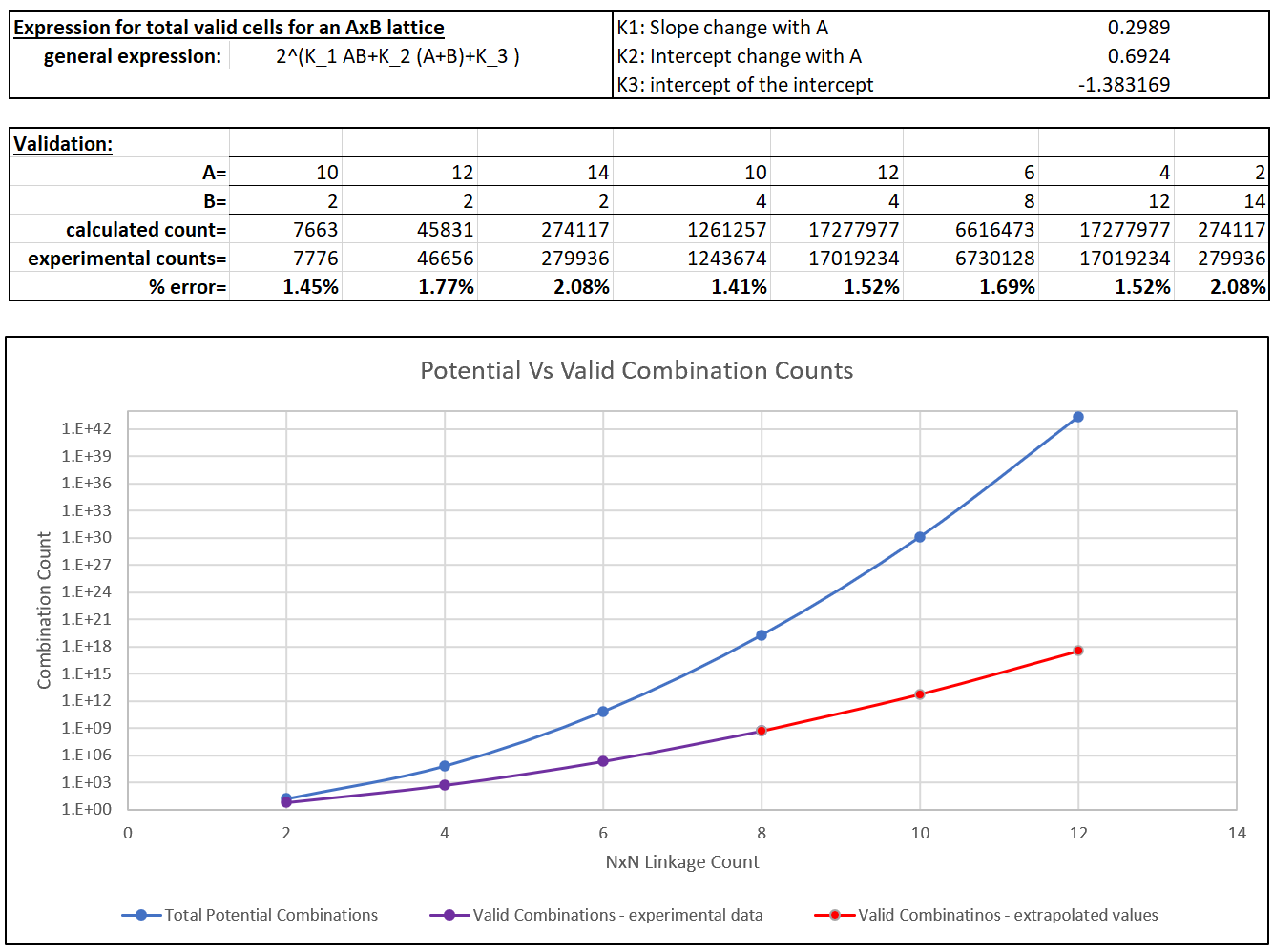

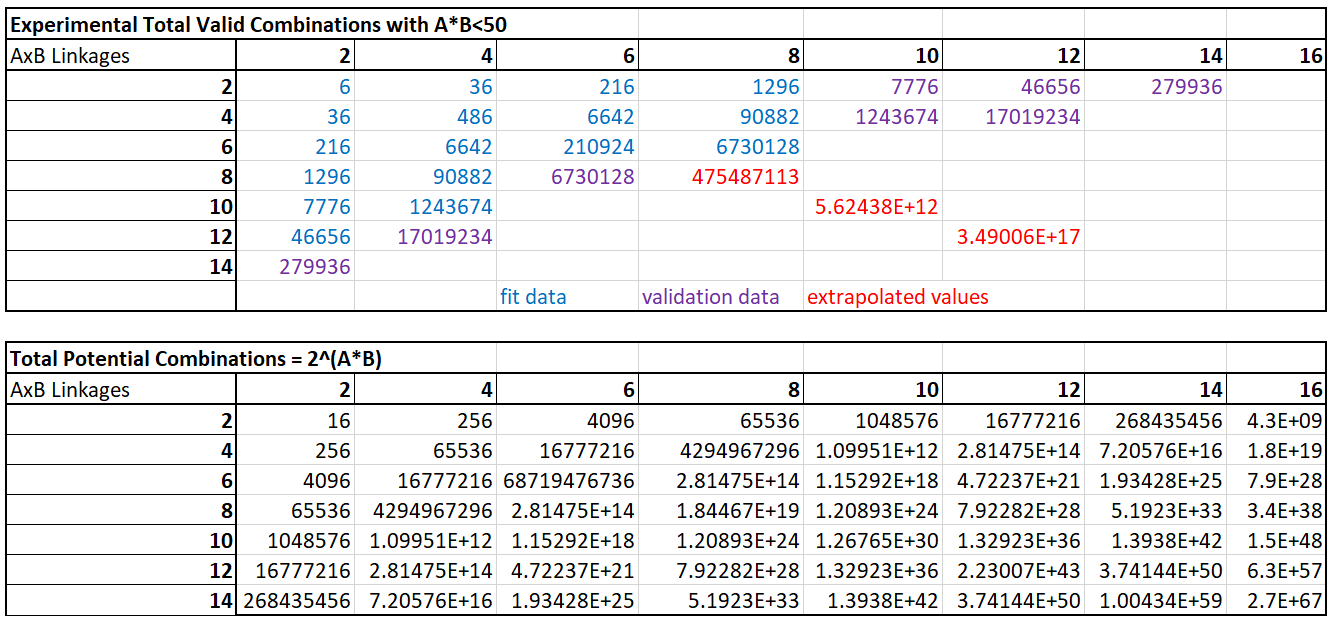


**S5. Programming and disturbance energy. A)** Force vs displacement plots for an inwards trajectory TCM cell at four different levels of compression. **B)** Force vs displacement plots for an outwards trajectory TCM cell at four different levels of compression. **C)** Force vs displacement plots for a shear trajectory TCM cell at four different levels of compression. D) Force vs displacement plots BEUs at seven different levels of tension.


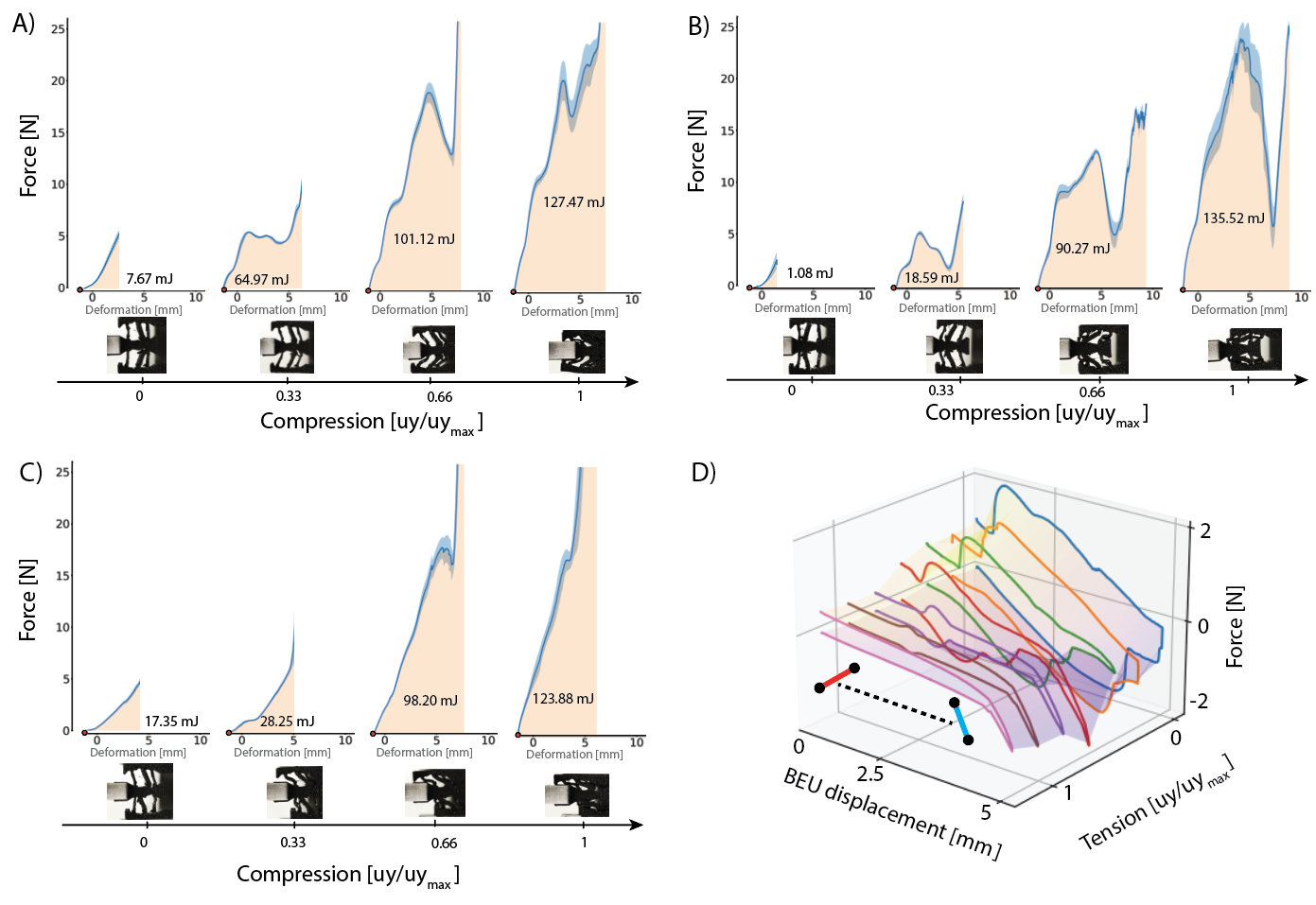


**S6. Shape processing.** We pre-process general shapes, converting them into left and right profile functions that act as inputs to the shape matching algorithm.


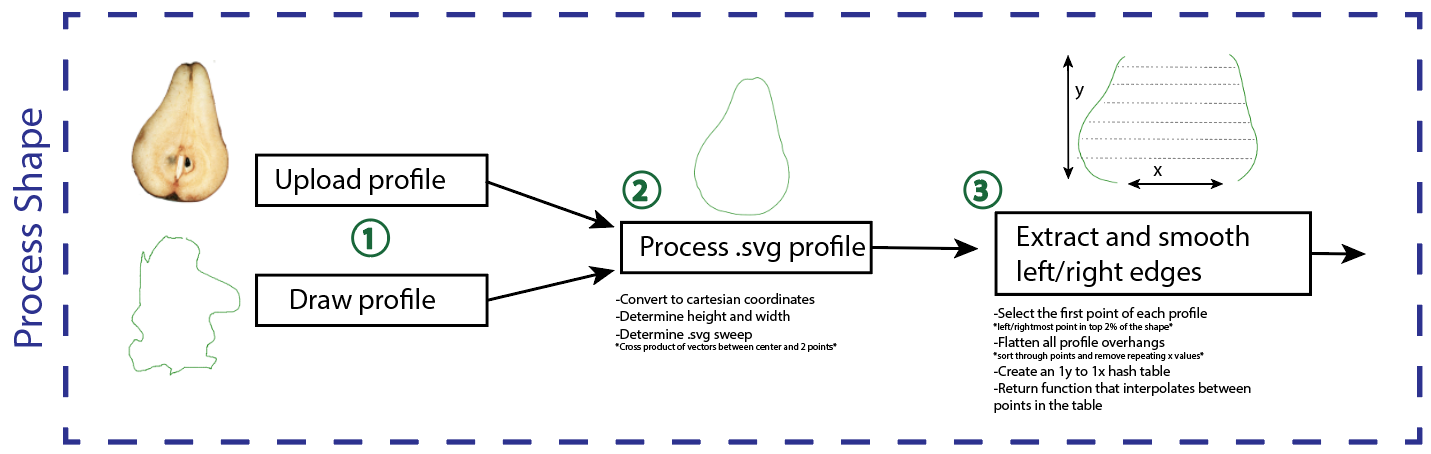


**S9. Error between physical tests and simulated profiles in expressed states. A)** Error between simulated and physically expressed profiles for both left-side and right side of the beaker shape. **B)** Error between simulated and physically expressed profiles for both left-side and right side of the face shape.


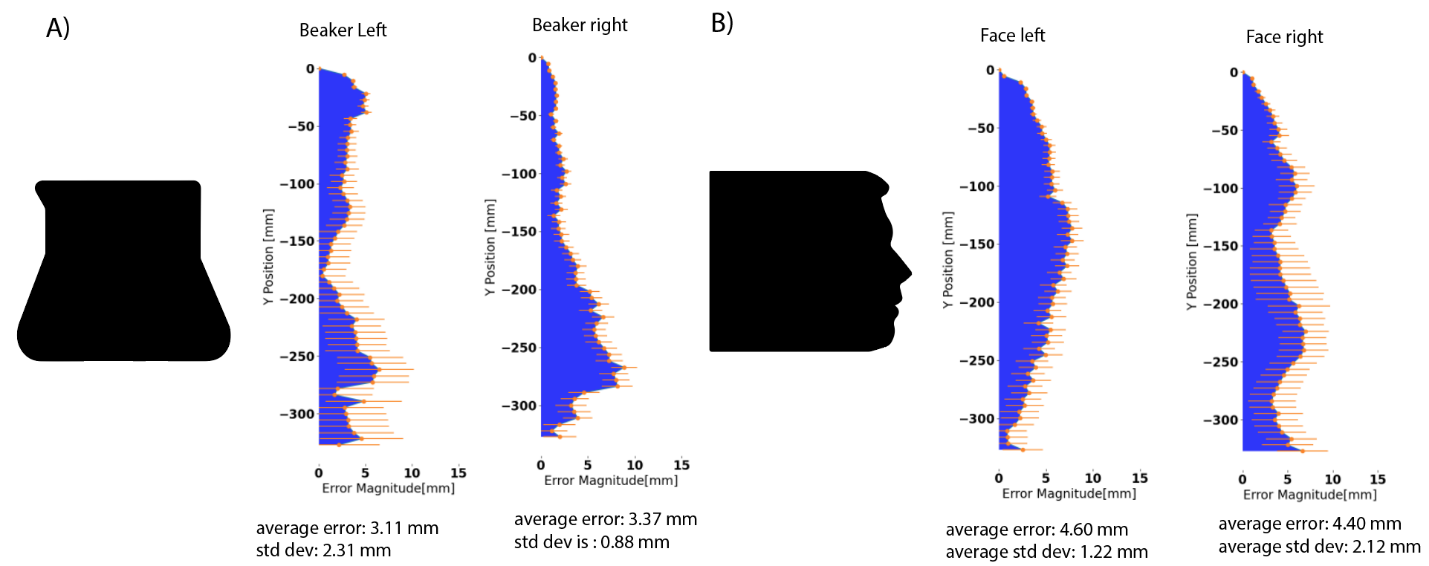


**S8. Physically programming the TCM.** To automatically adjust the physical encoding of a TCM, we generate G-Code, run it on our custom programming machine, and express the structure with a compressive force.


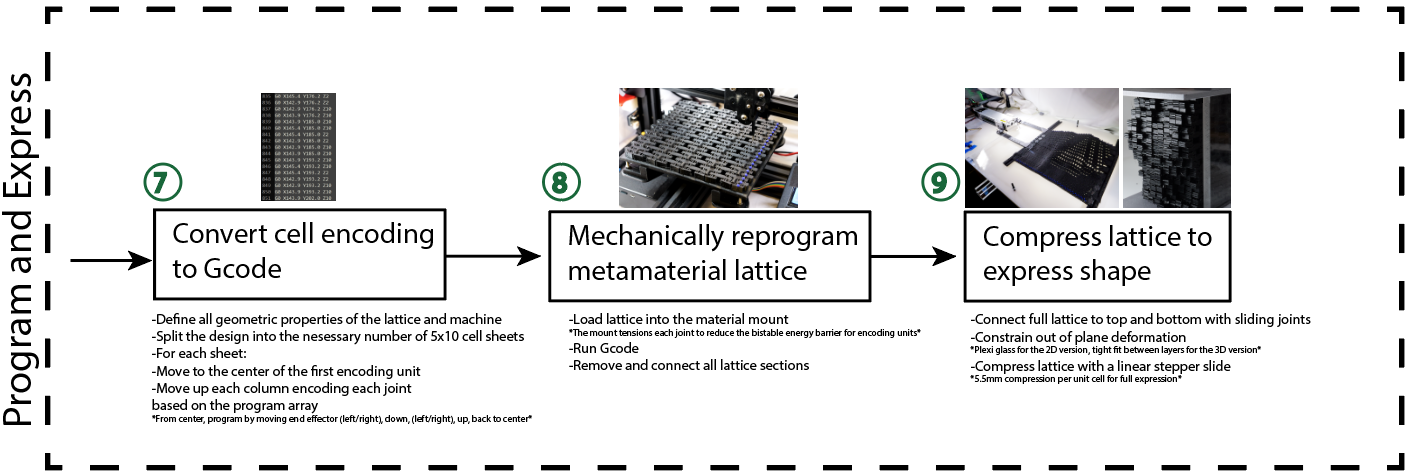


**S7. Valid encoding generation.** To encode TCM to match a desired profile trajectory, we approximate the profile of each side with discrete line segments and fill in interior cells to create a valid configuration.


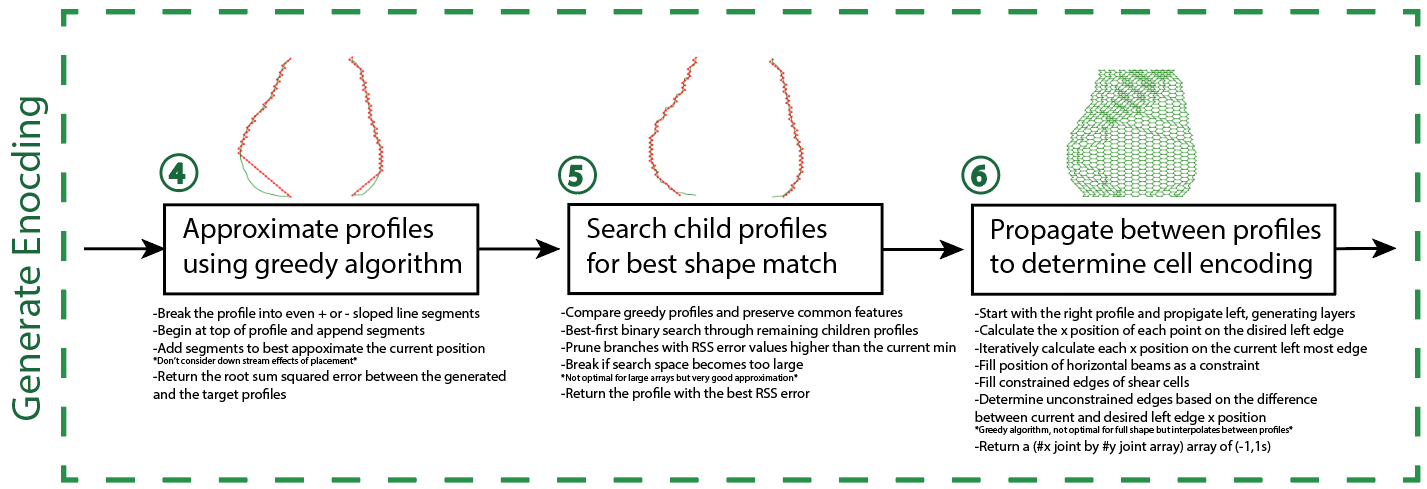


**Modeling of Reconfiguration Dynamics and Programming Speed**

To evaluate the scalability of Transition-Controlled Metamaterials (TCMs), we model the reconfiguration process kinematically and temporally. Consider an N x M array of bistable encoding units (BEUs), where N is the number of rows along the material transport axis and M is the number of columns. The total operational time decomposes into material transport and shape programming: $T_{total} = T_{trans} + T_{prog}$.

The time for transport( $T_{trans}$) is the time the lattice must physically travel through the loading and unloading sequence. With$L_{c}$the characteristic length of a BEU along the transport axis and $v_{load}$ the feed velocity, and assuming symmetric loading and unloading, the transport time scales linearly with N: $T_{trans} = T_{unload} + T_{load} = 2 (N \frac{L_{c}}{v_{load}})$. This imposes a fundamental O(N) bound: even with instantaneous programming, the maximum reconfiguration speed is limited by material-handling kinematics.

For the programming time there is, however, a serial versus parallel programming tradeoff possible. In the current laboratory implementation, a single end-effector addresses each BEU individually. With $t_{p}$ the actuation time to flip one unit and $t_{transit}$ the toolhead travel time between adjacent units, the programming time scales with lattice area: $T_{prog,serial} = N M (t_{p} + t_{transit})$. The O(N M) term dominates as resolution increases. In the current prototype, the measured current prototype programming rate is approximately 1 s per BEU, and the measured programming time scales directly with the number of BEUs addressed. Because BEUs are mechanically decoupled during programming, they need not be processed sequentially. In a fully parallel architecture all units could be actuated simultaneously and the programming time becomes independent of lattice size: $T_{prog,parallel} = t_{p}$, giving $T_{parallel} = 2 (N \frac{L_{c}}{v_{load}})+ t_{p}$. Under parallel programming the O(N M) bottleneck is eliminated and the reconfiguration time is bounded only by O(N) material transport. The serial speed of the current prototype is therefore an implementation choice rather than a fundamental limit; $t\_p$ can be reduced by lowering the unloading force and by scaling the lattice down so that switching distances shrink.

The per-cell programming energy follows from the single-BEU measurements reported in the main text. At full extension each BEU requires about 1.27 mJ to program (7.88 mJ in the neutral state), so a four-BEU cell requires roughly 5.1 mJ at full extension (about 31.5 mJ in the neutral state). Programming energy scales with the number of cells and is independent of whether programming is serial or parallel; only the time differs.

**Effective Stiffness of an N x M Lattice**

We model the lattice as a grid of identical linear springs of stiffness k, with N parallel columns and M springs in series per column. Series combination within a column gives $k_{column} = k / M$, and parallel combination of N columns gives the effective stiffness $k_{eff} = (N / M) k$. For a square network (N = M) the effective stiffness reduces to $k_{eff} = k$, so the global stiffness is independent of size when the parallel and series counts are balanced. This model links the single-cell compression measurements below to the global compression force and energy of a lattice of any size and composition.

**Compression Force by Cell State**

We measured the force-displacement response of single TCM cells in each programmed state, using an Instron Universal Testing System. The force required to reach 6 mm of compression and the resulting effective stiffness vary systematically with cell state. These varied primarily because of the cell construction The cells were printed in the auxetic state. The positive shear, and zero Poisson’s ratio states cause the BEU selector to be pre-buckled reducing the final effective stiffness and total force. The values are in Table S1 and the force displacement curves are in Figure S.10. We extracted the effective stiffness using the secant method.


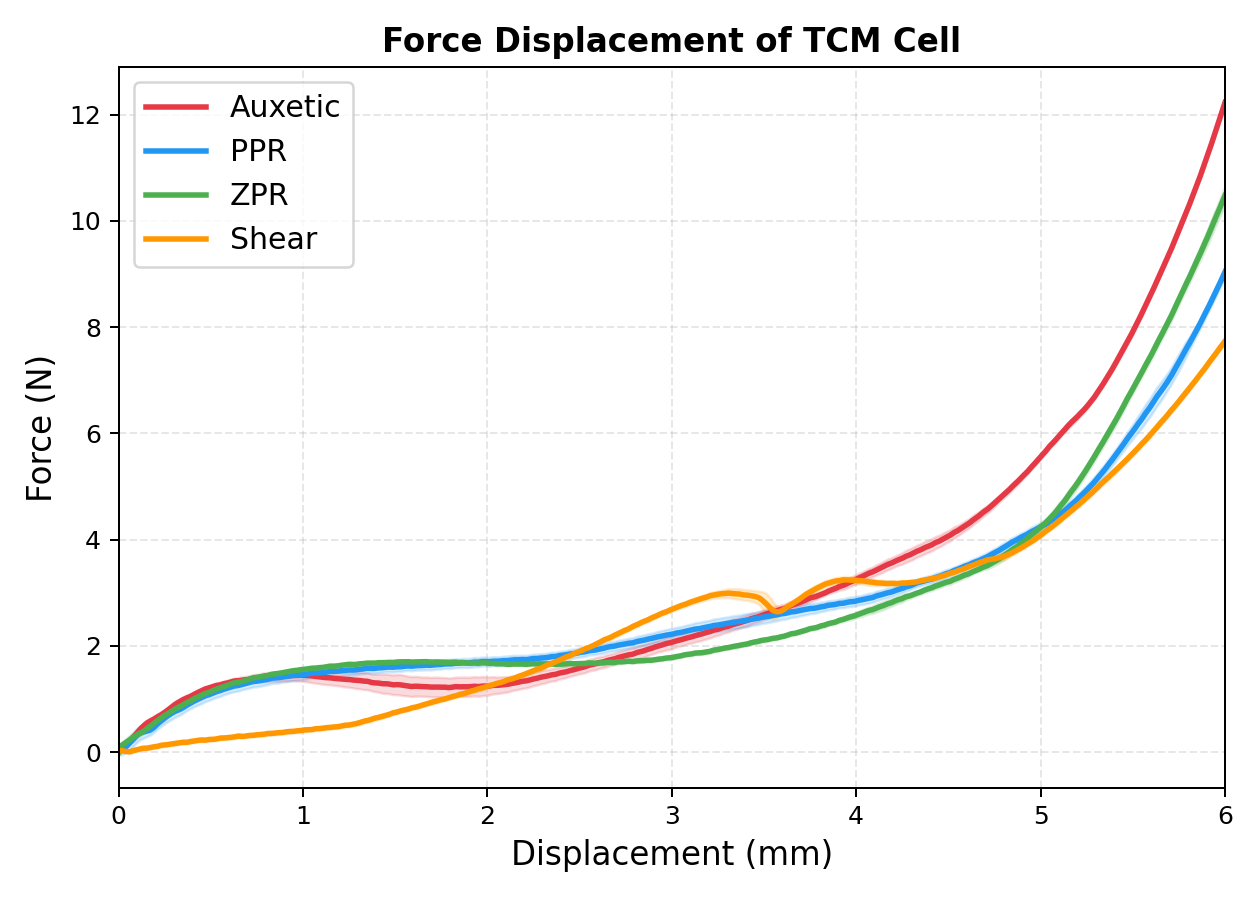


**Figure S.10:** Force-displacement response of a TCM cell in the auxetic, positive-Poisson-ratio (PPR), shear, and zero-Poisson-ratio (ZPR) states; shaded bands show the standard deviation across samples.

| **Cell State** | **Force at 6mm compression (N)** | **Effective Stiffness (secant)** |
| --- | --- | --- |
| Auxetics | 12.23 +/- 0.05 | 2.038 ± 0.008 |
| Positive Poisson ratio | 9.04 +/- 0.09 | 1.507 ± 0.016 |
| Zero Poisson ratio | 10.48 +/- 0.17 | 1.746 ± 0.028 |
| Shear | 7.74 +/- 0.04 | 1.289 ± 0.007 |
| **Table S1:** Force and effective stiffness at full compaction | | |

**Profile Approximation Error**

To quantify how closely a simulated expression reproduces a target profile, we added a normalized root-mean-square (RMS) error metric to our profile analysis. The point-wise deviation between the target and simulated profiles is normalized by the resolution and scaled by the shape height, then reported as a percentage of the shape width. For two representative targets, the left-side and right-side profile errors were 0.48% and 1.95%, and 0.65% and 0.68% of the shape width, respectively. Combined with the simulation-to-physical strain error reported in the main text (RMS strain error 0.0105), these results bound the overall target-to-physical agreement.


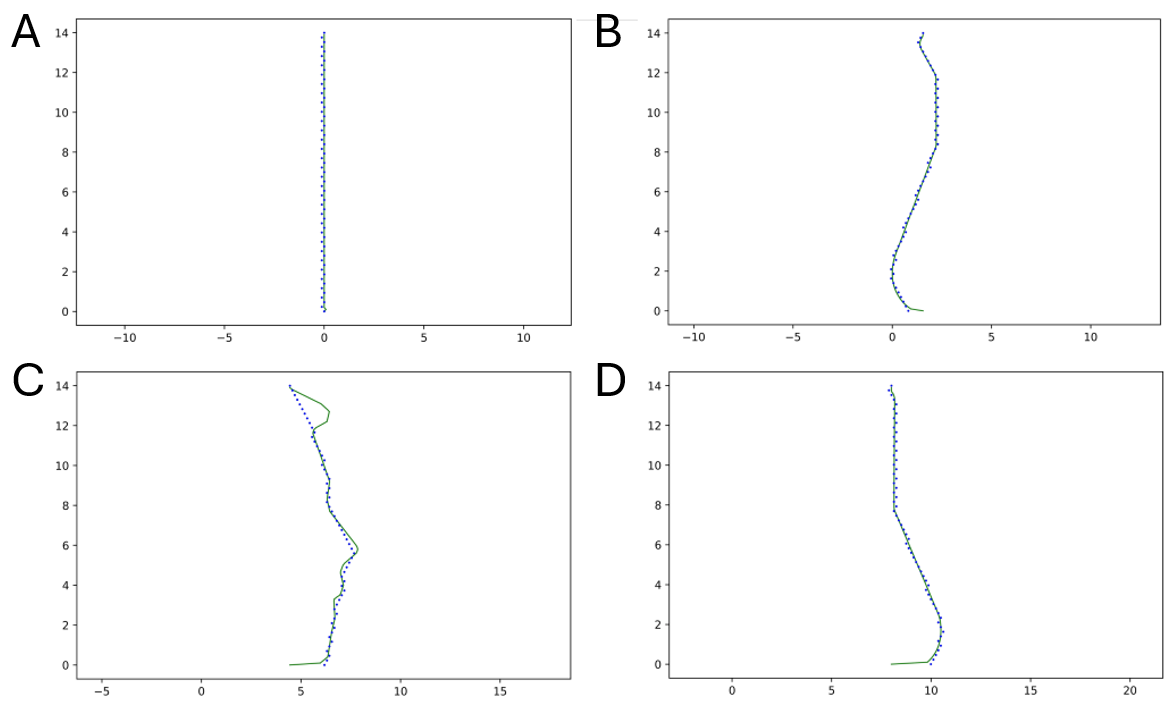
 **S.11 Comparison of target shape, and simulated shape matching.** The target (solid) and simulated (dotted) left- and right-side profiles for two representative shapes, with the corresponding normalized RMS errors. Left profiles are on the top and right profiles are on the bottom.

**References**

1. Ren, X., Das, R., Tran, P., Ngo, T. D. & Xie, Y. M. Auxetic metamaterials and structures: a review. *Smart materials and structures* **27**, 023001 (2018).

2. Li, D., Dong, L. & Lakes, R. S. A unit cell structure with tunable Poisson’s ratio from positive to negative. *Materials Letters* **164**, 456–459 (2016).

3. Berwind, M. F., Kamas, A. & Eberl, C. A hierarchical programmable mechanical metamaterial unit cell showing metastable shape memory. *Advanced Engineering Materials* **20**, 1800771 (2018).

4. Jin, L. *et al.* Guided transition waves in multistable mechanical metamaterials. *Proceedings of the National Academy of Sciences* **117**, 2319–2325 (2020).

5. Wang, H., Lu, Z., Yang, Z. & Li, X. In-plane dynamic crushing behaviors of a novel auxetic honeycomb with two plateau stress regions. *International Journal of Mechanical Sciences* **151**, 746–759 (2019).

6. Wang, Z. & Hu, H. Auxetic materials and their potential applications in textiles. *Textile Research Journal* **84**, 1600–1611 (2014).

7. Qiao, J. X. & Chen, C. Q. Impact resistance of uniform and functionally graded auxetic double arrowhead honeycombs. *International Journal of Impact Engineering* **83**, 47–58 (2015).

8. Duncan, O. *et al.* Review of auxetic materials for sports applications: Expanding options in comfort and protection. *Applied Sciences* **8**, 941 (2018).

9. Lipton, J. I. *et al.* Handedness in shearing auxetics creates rigid and compliant structures. *Science* **360**, 632–635 (2018).
